# Dietary inflammatory index and cardiovascular risk and mortality: an updated systematic review and meta-analysis

**DOI:** 10.3389/fcvm.2025.1626523

**Published:** 2025-11-20

**Authors:** Yu Ni, Qian Yao, Ting Xu, Xiuchuan Li

**Affiliations:** 1 School of Nursing, Chengdu Medical College, Chengdu, China; 2 Department of Emergency, Chengdu Third People’s Hospital, Chengdu, China; 3 School of Nursing, Chengdu University of Traditional Chinese Medicine, Chengdu, China; 4 Department of Cardiology, General Hospital of the Western Theater of the Chinese People’s Liberation Army, Chengdu, China

**Keywords:** cardiovascular diseases, dietary inflammatory index, risk, mortality, meta-analysis, updated systematic review

## Abstract

**Background:**

Cardiovascular diseases (CVDs) are the leading cause of death globally, and chronic inflammation is pivotal in CVDs development. Pro-inflammatory diets may exacerbate inflammation and thus increase CVDs risk. The Dietary Inflammatory Index (DII) is a validated measure of the inflammatory potential of diet. This updated systematic review and meta-analysis was conducted to clarify the association between DII and CVDs incidence and mortality.

**Methods:**

A comprehensive search was conducted in Pub Med, Web of Science, Embase, Cochrane Library, and China National Knowledge Infrastructure (CNKI) until February 2025. Study quality was assessed using the Newcastle-Ottawa Scale (*N*OS). Risk ratios (HR) and 95% confidence intervals (CI) were pooled using Review Manager 5.4, with subgroup analyses performed. Sensitivity and publication bias analyses were conducted using Stata 18.0.

**Results:**

Thirty cohort studies (NOS ≥7) from nine countries, involving 669,205 participants, were included. Compared with the lowest DII category, the highest category was associated with increased risks of CVD incidence [HR = 1.23, 95% CI (1.14–1.33); *I*^2^ = 54%] and mortality [HR = 1.29, 95% CI (1.24–1.35); *I*^2^ = 16%]. Stratified analyses indicated higher incidence risk among men (HR = 1.51) and higher mortality risk among women (HR = 1.25). Subgroup analyses further revealed a significant positive association between elevated DII and myocardial infarction (HR = 1.41). In models stratified by diabetes history, unadjusted associations were stronger (HR = 1.40), while adjusted associations were attenuated but remained significant, with a significant interaction (*P* = 0.002). Sensitivity and trim-and-fill analyses confirmed the robustness of these associations (all *P* < 0.001).

**Conclusion:**

Higher DII scores, reflecting pro-inflammatory dietary patterns, are significantly associated with increased risks of CVD incidence and mortality. These findings underscore the clinical and public health importance of promoting anti-inflammatory dietary strategies to mitigate the global CVD burden.

**Systematic Review Registration:**

https://www.crd.york.ac.uk/PROSPERO/view/CRD420250654615, PROSPERO, CRD420250654615.

## 1 Introduction

With the continuous intensification of societal aging and the significant increase in the consumption of ultra-processed foods, the incidence and mortality rates of cardiovascular diseases (CVDs) have been showing a rising trend (1). The Global Burden of Disease Study 2021 (GBD 2021) reported that between 1990 and 2021, the number of new CVD cases rose from 34.74 million to 66.81 million, while deaths increased from 12.33 million to 19.42 million. Although age-standardized incidence and mortality rates declined overall, absolute numbers grew substantially, with marked regional disparities (2) 2021, dietary risk factors were linked to 6.58 million CVD deaths, highlighting the considerable potential of dietary prevention and intervention to reduce the global burden (1). Moreover, evidence from multiple countries indicates that exposure to ultra-processed foods is independently associated with elevated CVD risk, further emphasizing the interplay among diet quality, inflammation, and cardiovascular health, and their public health implications (3, 4).

Based on previous research findings, the pathogenesis of cardiovascular diseases is extremely complex, involving a process of multi-factor interaction and multi-mechanism synergistic effects. In this complex pathological process, chronic inflammatory responses play a key role (5). Inflammatory biomarkers such as C-reactive protein (CRP), interleukin-6 (IL-6), and tumor necrosis factor-α (TNF-α) not only promote the formation of atherosclerotic plaques but also promote plaque instability and thrombosis, thereby triggering acute cardiovascular events (6) a modifiable factor of inflammation, diet's role mechanism has been increasingly focused on. Studies have shown that daily diet can directly influence the body's inflammatory state and can also establish a close link with cardiovascular diseases through the gut microbiota (7). As the “second genome” of humans, the composition and function of the gut microbiota are significantly influenced by daily dietary patterns (8). Long-term consumption of a diet rich in pro-inflammatory nutrients alters the gut microbiota structure, leading to impaired gut barrier function, which in turn triggers chronic inflammatory responses in the body, increasing the risk of cardiovascular disease incidence and mortality, In contrast, diets rich in *ω*-3 polyunsaturated fatty acids and polyphenols, which are anti-inflammatory nutrients, provide metabolic substrates for beneficial gut bacteria, promoting their proliferation and the production of short-chain fatty acids and other substances. Previous studies have shown that these substances not only regulate immune function, alleviate inflammation, but also improve endothelial cell function and reduce cardiovascular disease risk (9).

With the continuous deepening and expansion of nutritional science, the Dietary Inflammatory Index (DII), a tool designed to reflect the pro- or anti-inflammatory properties of diet, emerged. Constructed by researchers at the University of South Carolina through analyzing and integrating human, animal, and cell experiments, it includes 45 dietary nutrients and 6 inflammatory markers. To reflect specific nutrients' impacts on body inflammatory markers, the authors weighted related studies, assigned each dietary nutrient an inflammatory effect score via different weights, and calculated the impact of an individual's overall diet on body inflammation (10).

Currently, the DII has been widely used to explore links between diet and various inflammation—related diseases (especially cancer, digestive tract diseases, and cardiovascular diseases), becoming a research hotspot in recent years (11). Although existing evidence supports the DII-CVDs association (12), cohort studies published in the past five years have not been systematically evaluated. Thus, conducting a comprehensive and timely Meta-analysis is essential to provide a medical evidence—based basis for constructing precise dietary intervention strategies.

## 2 Methods

The reporting of this study followed the Preferred Reporting Items for Systematic Reviews and Meta-Analyses (PRISMA) guidelines (13), with the protocol registered on PROSPERO (ID: CRD420250654615).

### 2.1 Search strategy

We systematically searched PubMed, Web of Science, Embase, Cochrane Library, CNKI, Wanfang Data, and VIP databases for studies investigating the association between the DII and the risk of CVDs incidence or mortality. The search timeframe spanned from database inception to February 2025. To minimize omissions, we manually reviewed the reference lists of relevant articles. The detailed search strategy is provided in Supplementary Material 1.

### 2.2 Study selection

Table 1 outlines the PICOS criteria for study inclusion. Studies meeting the following criteria were eligible: (1) Study type: Cohort studies (retrospective or prospective); (2) Population: Adults aged ≥18 years; (3) Exposure: DII as the primary exposure variable; (4) Outcome: Incident CVDs or CVDs-related mortality; (5) Statistical reporting: Full effect estimates, including hazard ratios (HRs) with 95% confidence intervals (CIs). Exclusion criteria included: (1) Non-original research (e.g., reviews, conference abstracts, book chapters) or secondary evidence (e.g., systematic reviews); (2) Unavailable full-text articles; (3) Duplicate publications; (4) Insufficient data for extraction or conversion; (5) Low methodological quality. Two investigators (YN and QY) independently performed study selection, with discrepancies resolved through team discussions.

**Table 1:** Picos criteria of this study.

PICOS element	Description
P	Adults
I	Dietary Inflammatory Index
C	Highest vs. lowest DII quantiles
O	CVDs incidence or mortality
S	cohort studies (retrospective or prospective)

PICOS, participant, intervention, comparison, outcome, and study design.

### 2.3 Data extraction

To ensure comprehensive data extraction, two investigators (YQ and XT) independently performed data extraction using a predefined standardized template. Any discrepancies encountered during the process were resolved through team discussions. Extracted data included: Study characteristics (first author, publication year, study location, study design, follow-up duration); Participant information (age, sex ratio, health status); Assessment methods (dietary survey methods, dietary evaluation tools, criteria for ascertaining CVDs incidence and mortality); Statistical analyses: Hazard ratios (HR) with 95% confidence intervals (CI) comparing the highest vs. lowest DII quantiles.

### 2.4 Quality assessment

Two investigators (YN and TX) independently evaluated study quality using the Newcastle-Ottawa Scale (NOS), a validated tool developed by the Ottawa Hospital Research Institute for assessing observational studies. The NOS comprises three domains: participant selection (4 items), comparability of groups (1 item), and outcome assessment (3 items), with a maximum score of 9. Studies scoring 7–9 were classified as high quality with low overall bias and included in the systematic review and meta-analysis. Studies scoring ≤6 were excluded due to elevated risk of bias. Discrepancies in scoring were resolved through team consensus.

### 2.5 Statistical analysis

Data were analyzed using Stata 18.0 and Review Manager 5.4. Pooled HRs with 95% CIs were calculated to evaluate associations between DII and CVDs incidence/mortality. Heterogeneity was assessed using Cochran's *Q*-test and the *I*^2^ statistic (significance level *α* = 0.1). A fixed-effect model was applied if *I*^2^ < 50% and *P* > 0.1; otherwise, a random-effect model was used. Descriptive analyses were conducted when insufficient data precluded meta-analysis. Subgroup analyses explored heterogeneity sources, sensitivity analyses assessed result robustness, and funnel plots with trim-and-fill adjustments evaluated publication bias.

## 3 Results

**Figure 1:**
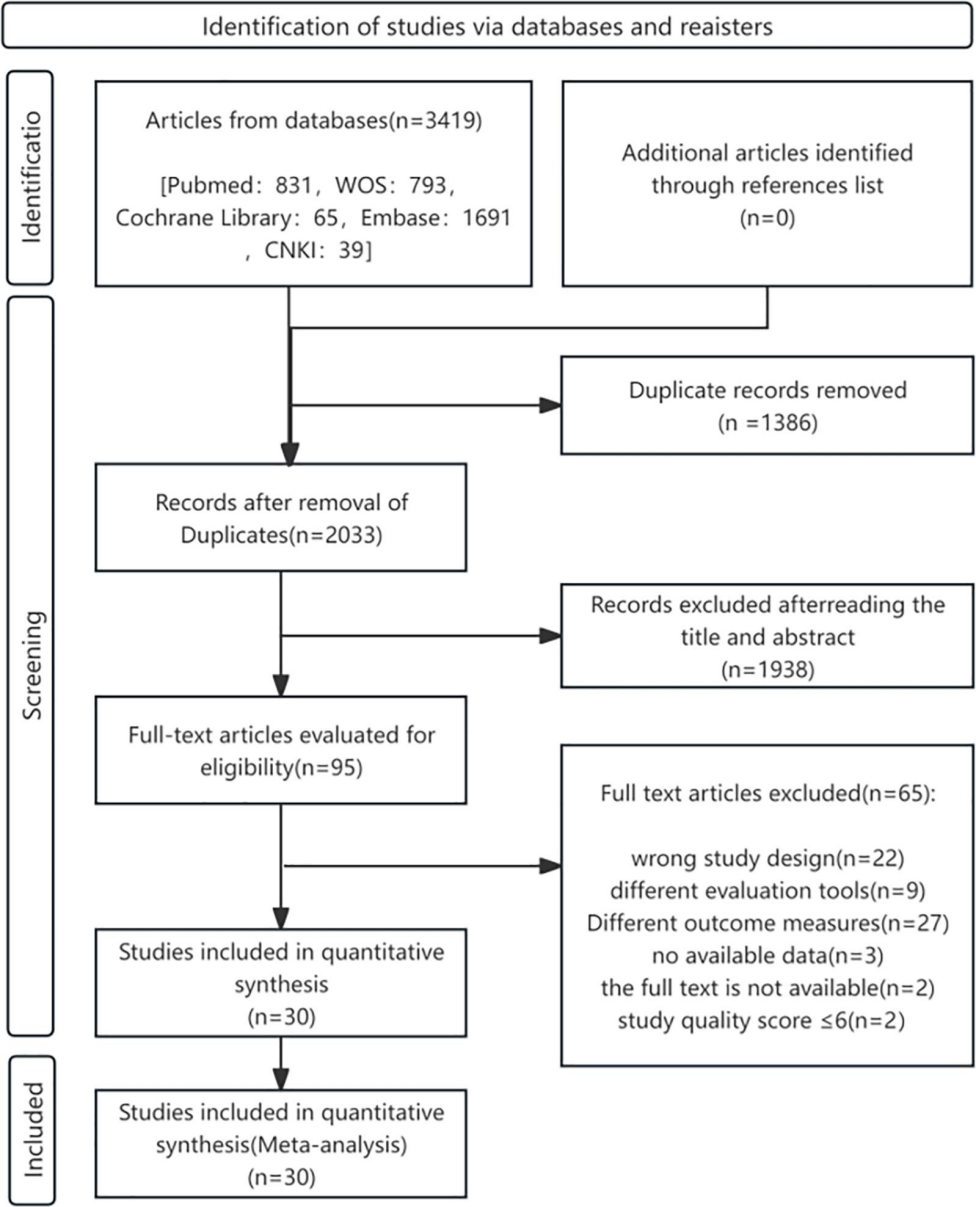
Flowchart of study selection process for the meta-analysis of DII and CVDs risk/mortality.

Figure 1 outlines the literature screening process. Initial database searches yielded 3,419 records. After excluding 1,386 duplicates and 1,938 irrelevant studies through title/abstract screening, 95 full-text articles were reviewed. Ultimately, 30 English-language cohort studies met the inclusion criteria.

All the studies mentioned above were evaluated using the Newcastle-Ottawa Scale (NOS), with all scores ≥7, encompassing 669,205 participants across nine countries (14–43). The basic characteristics and quality assessments of studies investigating DII—CVDs incidence and mortality are presented in Tables 2, 3, respectively.

**Table 2:** Basic characteristics and quality assessment of studies on the risk of CVDs associated with DII.

First author	Year of publication	Country	Study type	Follow-up duration (years)	Population	Age (years)	Sample size	Number of incident cases (n)	Dietary assessment method	Outcome measures	Covariates	Quality Assessment
Asadi et al. (14)	2019	Iran	Cohort study	6	Normal adults	35–65	4,672	①124/②24	FFQ	①②	Adjusted for age, energy, BMI, physical activity level, education level, marital status, and smoking status.	7
Ramallal et al. (19)	2015	Spain	Cohort study	8.9	College graduates	38 ± 12	18,794	①117	FFQ	①	Age, sex, cardiovascular risk factors, total energy intake, physical activity, BMI (BMI), educational level, other cardiovascular diseases, baseline special diet, snacking, average sedentary time, average television viewing time	7
Khan et al. (16)	2020	Korea	Cohort study	7.4	Normal adults	40–79	1,62,773	①1,111/②824/⑤288	SQ-FFQ	①②⑤	Age, smoking, alcohol consumption, physical activity, BMI, and energy intake	9
Liu et al. (17)	2024	Chain	Cohort study	18	Normal adults	40.8 ± 12.0	4,822	①234/②114/⑤136	24HR	①②⑤	Age, sex, residential area, smoking status, educational level, physical activity, alcohol intake, hypertension, diabetes, and BMI	7
Vissers et al. (21)	2016	Australia	Cohort study	11	Healthy adult women	52.0 ± 1	6,972	①335/②69/④191/⑤40	FFQ	①②④⑤	Age, energy level, diabetes, hypertension, smoking status, educational level, menopausal status, physical activity, and alcohol consumption	7
Francisc et al. (15)	2020	Mexico	Cohort study	11.1	HCW	45.0 ± 12.3	339	③382	FFQ	③	Age, sex, smoking status, educational level, physical activity, sleep duration, and energy intake	7
Vissers et al. (22)	2017	Australia	Cohort study	12	Healthy adult women	52 ± 1	7,169	③1,681	FFQ	③	Age, energy level, diabetes, smoking status, educational level, menopausal status, physical activity, and BMI	7
Villaverde et al. (20)	2024	Mexico	Cohort study	5	HCW	45.6 ± 7.3	1,540	③341	FFQ	③	Age, sex, energy intake, physical activity, smoking, sleep, and educational level	8
MacDonald et al. (18)	2020	France	Cohort study	21	Healthy adult women	50.1 ± 6.3	46,652	③13,183	FFQ	③	Physical activity, smoking, family history of CVDs, educational level, diabetes, dyslipidemia, and BMI	7
Ze et al. (24)	2023	Chain	Cohort study	7	Normal adults	41.85 ± 13.94	10,694	③3,687	24HR	③	ge, sex, residential location, education, physical activity level, smoking, alcohol consumption, self-reported diabetes, BMI, waist circumference, baseline systolic and diastolic blood pressure, sodium-to-potassium intake ratio, and intakes of energy, fat, protein, and carbohydrates	8
Zuercher et al. (25)	2023	Spain	Cohort study	12.9	PMW	50–79	3,469	④118/⑤97	FFQ	④⑤	Age, sex, lifestyle-related risk factors, smoking, alcohol consumption, physical exercise, educational level, diabetes, hypertension, hypercholesterolemia, BMI, and socioeconomic status	7
Wu et al. (23)	2023	Chain	Cohort study	18	Normal adults	45 ± 15	14,652	②280/⑤404	24HR + WFR	②⑤	Age, sex, BMI, educational level, region, urbanization index, physical activity, baseline history of hypertension, smoking status, alcohol consumption, total energy intake	8
Garcia-Arellano et al. (41)	2015	Spain	Cohort study	4.8	Normal adults	67.0 ± 6.2	7,169	①227	24HR	①	Age and sex, overweight/obesity, waist-to-height ratio, total energy intake, smoking status, diabetes mellitus, hypertension, dyslipidemia, family history of premature cardiovascular disease, physical activity, and educational level.	7
Neufcourt et al. (42)	2016	France	Cohort study	11.4	Normal adults	49.1 ± 6.3	7,743	①292/②93/⑤58	24HR	①②⑤	sex and energy intake without alcohol, supplementation group, number of 24-h records, education level, marital status, smoking status, and physical activity, BMI	7

HCW, healthcare workers; PMW, postmenopausal women; ① = The occurrence of cardiovascular disease; ② = The occurrence of myocardial infarction; ③ = The occurrence of hypertension; ④ = The occurrence of coronary heart disease; ⑤ = The occurrence of stroke; FFQ, Food Frequency Questionnaire; SQ-FFQ, Semi-Quantitative Food Frequency Questionnaire; 24HR, 24-hour dietary recall; WFR, weighed food record; BMI, body mass index.

**Table 3:** Basic characteristics and quality assessment of studies on the risk of CVDs mortality associated with DII.

First author	Year of publication	Country	Study type	Follow-up duration (years)	Population	Age (years)	Sample size (n)	Number of death cases (n)	Dietary assessment method	Outcome measures	Covariates	Quality assessment
Choi et al. (27)	2024	USA	Cohort Study	18.7	Normal Adults/NWCO	46.4	1,777/1,744	①42/①166	24HR	①	Age, sex, race, ethnicity, income, educational level, physical activity level, and smoking status	8
Huang et al. (29)	2023	USA	Cohort Study	9	Hyperuricemic Patients	≥20	3,039	①254	24HR	①	Sex, age, race, educational level, PIR, smoking history, alcohol history, hypertension, diabetes, eGFR, serum cholesterol, and serum triglycerides	7
Ma et al. (30)	2025	USA	Cohort Study	9.5	MetS	53.74 ± 0.22	13,751	①639	24HR	①	Age, sex, race, educational level, BMI, PIR	7
Majidi et al. (31)	2024	USA	Cohort Study	30	Normal Adults	46.90 ± 13.1	1,440	①118	FFQ	①	Smoking status and medical conditions (self-reported diabetes, dyslipidemia, hypertension, angina, heart attack, stroke, cancer, and medication use for heart disease or diabetes), education, BMI, physical activity, and use of dietary supplements	7
Okada et al. (32)	2019	Japan	Cohort Study	19.3	Normal Adults	40–79	1,10,585	①1,524/②4,248	FFQ	①②	Age, geographic region, BMI, educational level, smoking status, exercise habits, sleep duration, history of hypertension and diabetes, and total energy intake	7
Park et al. (33)	2018	USA	Cohort Study	18.2	Normal Adults	45–75	1,50,405	①3,292	Q-FFQ	①	Age, race/ethnicity, body mass index (BMI), history of diabetes, educational level, marital status, physical activity, alcohol intake, smoking status, energy intake, and use of menopausal hormone therapy (women only)	8
Nitin et al. (34)	2017	USA	Cohort Study	13.5	Normal Adults	>19	12,366	①1,233	24HR	①	Age, sex, race, diabetes status, hypertension, physical activity, BMI, PIR, and smoking	8
Sun et al. (35)	2023	USA	Cohort Study	6.7	Healthy Older Adults	73.29 ± 0.10	10,827	①1,230	FFQ	①	Age, sex, race, BMI level, educational level, PIR, smoking status, alcohol status, physical activity, total energy intake, and history of hypertension, diabetes, dyslipidemia, and cardiovascular disease	7
Veronese et al. (36)	2020	Italy	Cohort Study	12	Normal Adults	65.50 ± 9.5	1,565	①102	FFQ	①	Age, sex, smoking status, diabetes, gastric ulcer, gallstone, liver cirrhosis, cancer, acute myocardial infarction, BMI, systolic and diastolic blood pressure, presence of depressive symptoms, presence of hepatic steatosis, energy and alcohol intake, and adherence to the Mediterranean diet	7
Yang et al. (38)	2024	USA	Cohort Study	18	Normal Adults	40.8 ± 12.0	4,822	①755	24HR	①	Age, sex, educational level, race, BMI, smoking status, alcohol consumption, history of dyslipidemia, diabetes, hypertension, and levels of HbA1c, alanine aminotransferase (ALT), aspartate aminotransferase (AST), sodium, potassium, creatinine, total cholesterol, and high-density lipoprotein cholesterol (HDL-C)	7
Yuan et al. (39)	2022	USA	Cohort Study	12.9	Normal Adultsna	50–79	20,762	①243	FFQ	①	Age, sex, BMI, smoking, hypertension, educational level, dyslipidemia, recreational activity, and moderate or heavy alcohol consumption	8
Zhou et al. (40)	2024	USA	Cohort Study	6.6	Normal Adults	64.66 ± 0.31	5,006	①532	24HR	①	Marital status, survey cycle, educational level, PIR, BMI, smoking status, alcohol status, CPR, moderate recreational activity, hypertension, diabetes, coronary heart disease, and history of stroke	8
Deng et al. (28)	2016	USA	Cohort Study	11.25–14	Normal Adults/Prediabetes/T2MD	20–90	9,631/2,681/968	①676/①412/①240	24HR	①	Age, sex, race, HbA1c, current smoking, physical activity, BMI, systolic blood pressure (SBP)	7
Bondonno et al. (26)	2017	Australia	Cohort Study	15	PMW	75.1 ± 2.7	1,304	②269/③150	FFQ	②③	Age, BMI, energy intake, energy expenditure from physical activity, socioeconomic status, low-dose aspirin use, antihypertensive medication use, statin use, prevalent ASVD, and treatment	7
Xie et al. (37)	2024	USA	Cohort Study	17	Normal Adults	44.93	9,788	①668	24HR	①	Age, sex, race, waist circumference, physical activity, alcohol status, eGFR, high-sensitivity cardiac troponin T, high-sensitivity cardiac troponin I, HbA1c, Urine albumin-to-creatinine ratio, Low-density lipoprotein cholesterol, CRP, Triglycerides	7
Ganbaatar et al. (43)	2023	Japan	Cohort Study	29	Normal Adults	≥30	9,284	①1,149/②539/④234/⑤509	WFR	①②④⑤	Age, Sex, BMI, Smoking status, Alcohol consumption status, Work intensity, Energy-adjusted salt intake, Serum total cholesterol, Hypertension status, and Diabetes mellitus history.	8

NWCO, normal-weight central obesity; MetS, metabolic syndrome; T2MD, type 2 diabetes mellitus; PMW, postmenopausal women; ① = Mortality due to cardiovascular disease; ② = Mortality due to atherosclerotic cardiovascular disease; ③ = Mortality due to ischemic heart disease; ④ = The occurrence of coronary heart disease; ④ = The occurrence of stroke; FFQ, Food Frequency Questionnaire; 24HR, 24-hour dietary recall; Q-FFQ, Quantitative Food Frequency Questionnaire; ASVD, atherosclerotic vascular disease; WFR, weighed food record; BMI, body mass index; PIR, poverty income ratio; eGFR, estimated glomerular filtration rate; HbA1c, glycated hemoglobin; CRP, C-reactive protein.

### 3.1 Meta-Analysis results

#### 3.1.1 Association between DII and CVDs risk

**Figure 2:**
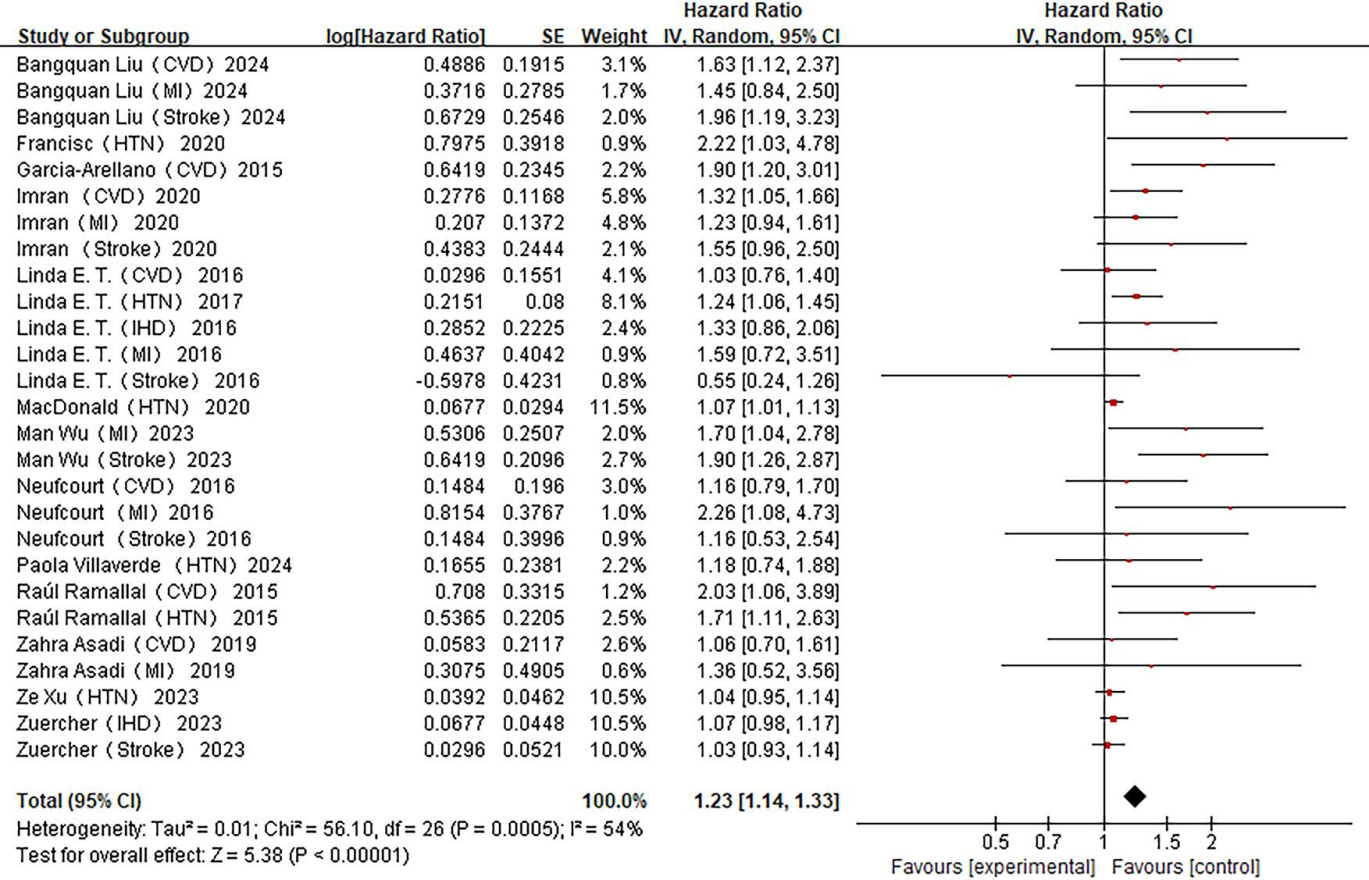
Forest plot of the association between DII and CVDs risk.

Fourteen studies examined the relationship between DII and CVDs incidence (14–25, 41, 42). Significant heterogeneity was observed across studies (*I*^2^ = 54%, *P* < 0.01), necessitating a random-effects model. The highest DII quantile was associated with a 23% increased risk of CVDs incidence compared to the lowest quantile [HR = 1.23, 95% CI (1.14–1.33)] (Figure 2).

Subgroup analyses stratified by outcome type, region, sex, dietary assessment method, BMI adjustment, energy adjustment, and diabetes history are summarized in Table 4. Between-group comparisons indicated significant effect modification by sex, dietary method, energy adjustment, and diabetes history. The strongest and most consistent association was observed for myocardial infarction [HR = 1.41, 95% CI (1.16–1.72)]. Associations were particularly evident among men [HR = 1.51, 95% CI (1.26–1.80)], studies using 24-hour dietary recall [HR = 1.53, 95% CI (1.19–1.94)], studies with energy adjustment [HR = 1.44, 95% CI (1.23–1.69)], and studies without diabetes history adjustment [HR = 1.40, 95% CI (1.25–1.56)].

**Table 4:** Subgroup analysis of the association between DII and CVDs risk.

Subgroup analysis	Number of studies included	Heterogeneity test		Effect model	Pooled effect size HR (95%CI)	Pooled effect size HR test		Intergroup heterogeneity
		*I*^2^ (%)	*P*			*Z*	*P*	*P*
Specific diseases								0.050
CVDs	7	44	0.110	Fixed	1.33 (1.15–1.53)	3.92	0.001	
HTN	6	57	0.040	Random	1.14 (1.03–1.26)	2.47	0.010	
MI	6	0	0.670	Fixed	1.41 (1.16–1.72)	3.42	<0.001	
IHD	2	0	0.340	Fixed	1.08 (0.99–1.18)	1.73	0.080	
Stroke	6	73	0.003	Random	1.31 (0.94–1.84)	1.60	0.110	
Study region								0.250
Europe	5	57	0.020	Random	1.26 (1.08–1.47)	2.68	0.007	
North America	2	47	0.170	Fixed	1.40 (0.94–2.08)	1.65	0.100	
Asia	5	45	0.080	Fixed	1.12 (1.04–1.21)	3.03	0.002	
Oceania	2	0	0.61	Fixed	1.21 (1.06–1.38)	2.90	0.004	
Gender								0.006
Male	3	0	0.610	Fixed	1.51 (1.26–1.80)	4.52	<0.001	
Female	7	32	0.130	Fixed	1.10 (1.05–1.15)	4.07	<0.001	
Dietary assessment method								0.020
24HR	4	74	<0.001	Random	1.53 (1.19–1.94)	3.29	0.01	
FFQ	8	35	0.090	Fixed	1.09 (1.04–1.13)	3.96	<0.001	
SQ-FFQ	1	N/A	N/A	N/A	N/A	N/A	N/A	
24HR + WFR	1	N/A	N/A	N/A	N/A	N/A	N/A	
Adjustment for BMI								0.670
Yes	10	55	0.001	Random	1.22 (1.12–1.31)	4.91	<0.001	
No	4	46	0.080	Fixed	1.25 (1.05–1.51)	2.44	0.010	
Adjustment for physical activity								N/A
Yes	13	55	<0.001	Random	1.24 (1.14–1.34)	5.33	<0.001	
No	1	N/A	N/A	N/A	N/A	N/A	N/A	
Adjustment for energy intake								0.005
Yes	8	60	0.002	Random	1.44 (1.23–1.69)	4.52	<0.001	
No	6	39	0.070	Fixed	1.11 (1.07–1.15)	4.02	<0.001	
Adjustment for diabetes history								0.002
Yes	7	54	0.010	Random	1.13 (1.05–1.22)	3.16	0.002	
No	7	0	0.490	Fixed	1.40 (1.25–1.56)	5.79	<0.001	

N/A, not applicable.

#### 3.1.2 Association between DII and CVDs mortality

**Figure 3:**
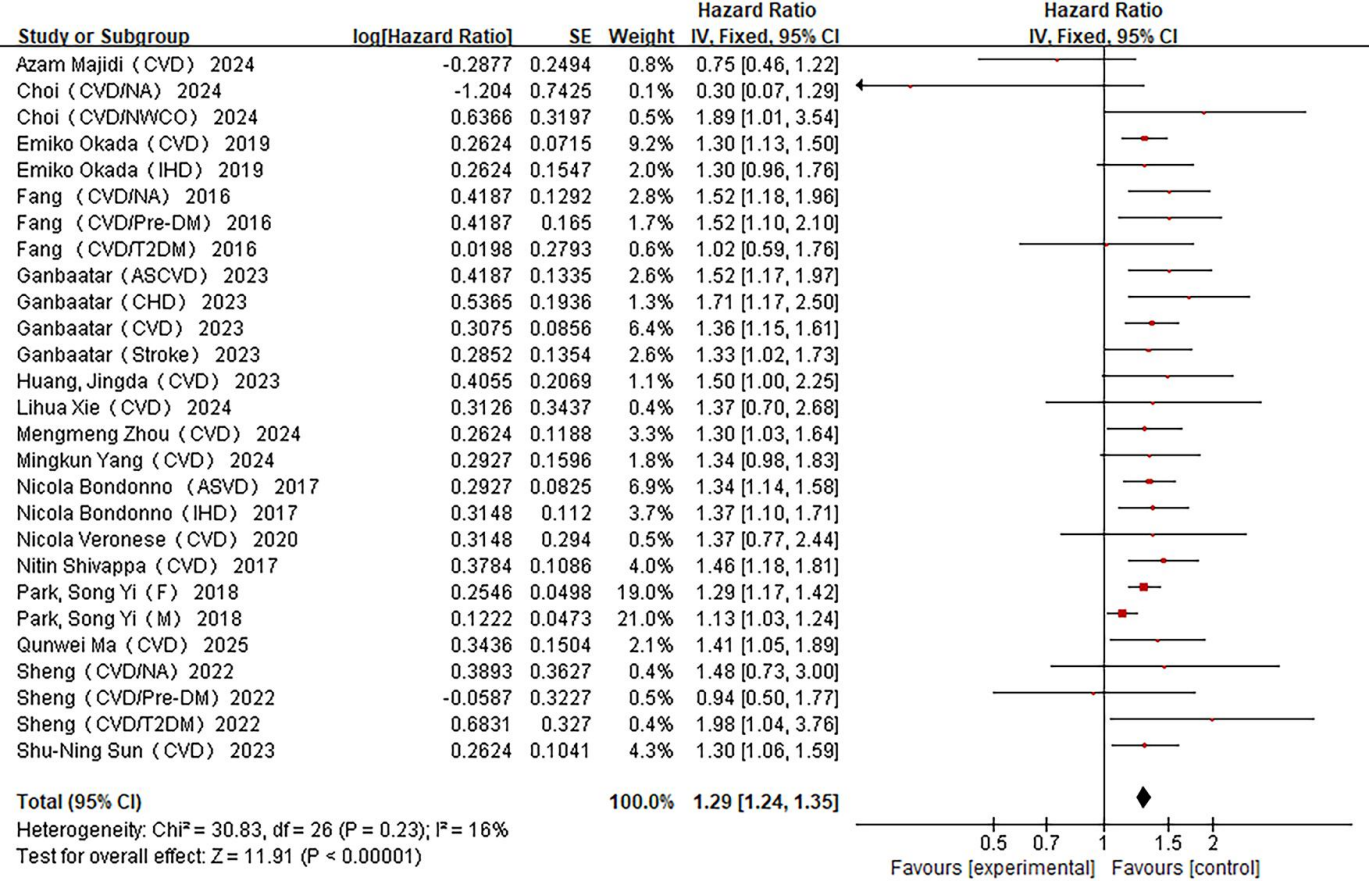
Forest plot of the association between DII and CVDs mortality.

Besides the above-mentioned studies, the remaining 16 studies assessed CVDs-related death events (26–40, 43). As shown in Figure 3, the meta-analysis revealed a positive association between the dietary inflammatory index and the risk of CVDs death [*I*^2^ = 16%, HR = 1.29, 95%CI (1.24–1.35)].

Similarly, subgroup analyses of the included studies were conducted based on factors such as disease status of the subjects, study region, gender, BMI adjustment, physical activity adjustment, dietary assessment method, energy intake adjustment, and diabetes history adjustment to explore the impact of each factor on the study results (Table 5). The subgroup—analysis results indicated that gender significantly contributed to heterogeneity (*P* < 0.05), with stronger associations observed in women [HR = 1.25, 95% CI (1.12–1.39)]. Other factors (disease status, region, dietary method, BMI adjustment, physical activity adjustment, energy adjustment, diabetes history) did not significantly explain heterogeneity.

**Table 5:** Subgroup analysis of the association between DII and CVDs mortality.

Subgroup analysis	Number of studies included	Heterogeneity test		Effect model	Pooled effect size HRHR (95%CI)	Pooled effect size HR test		Intergroup heterogeneity
		*I*^2^ (%)	*P*			*Z*	*P*	*P*
Disease status of study participants								0.200
Yes	5	0	0.530	Fixed	1.43 (1.22–1.68)	4.33	<0.001	
No	14	25	0.150	Fixed	1.28 (1.23–1.34)	10.97	<0.001	
Study region								0.740
USA	12	33	0.080	Fixed	1.26 (1.20–1.33)	8.62	<0.001	
Japan	2	0	0.760	Fixed	1.36 (1.25–1.48)	6.97	<0.001	
Other	2	0	0.990	Fixed	1.35 (1.19–1.53)	4.65	<0.001	
Gender								0.020
Male	6	0	0.900	Fixed	1.09 (1.05–1.14)	4.16	<0.001	
Female	8	74	<0.001	Random	1.25 (1.12–1.39)	4.09	<0.001	
Dietary assessment method								0.380
24HR	9	0	0.870	Fixed	1.39 (1.27–1.53)	7.15	<0.001	
FFQ	6	60	0.020	Random	1.18 (1.11–1.25)	3.50	<0.001	
Q-FFQ	1	N/A	N/A	N/A	N/A	N/A	N/A	
WFR	1	N/A	N/A	N/A	N/A	N/A	N/A	
Adjustment for BMI								0.830
Yes	13	12	0.300	Fixed	1.29 (1.24–1.35)	11.68	<0.001	
No	3	43	0.150	Fixed	1.45 (1.08–1.95)	2.84	0.004	
Adjustment for physical activity								0.150
Yes	11	34	0.110	Fixed	1.26 (1.20–1.32)	8.91	<0.001	
No	5	0	0.970	Fixed	1.41 (1.28–1.56)	6.82	<0.001	
Adjustment for energy intake								0.270
Yes	6	31	0.140	Fixed	1.27 (1.21–1.33)	9.79	<0.001	
No	10	0	0.500	Fixed	1.38 (1.26–1.52)	6.76	<0.001	
Adjustment for diabetes history								0.230
Yes	10	23	0.190	Fixed	1.27 (1.21–1.33)	9.94	<0.001	
No	6	0	0.530	Fixed	1.39 (1.26–1.52)	6.75	<0.001	

N/A, not applicable.

### 3.2 Sensitivity analysis and publication bias

**Figure 4:**
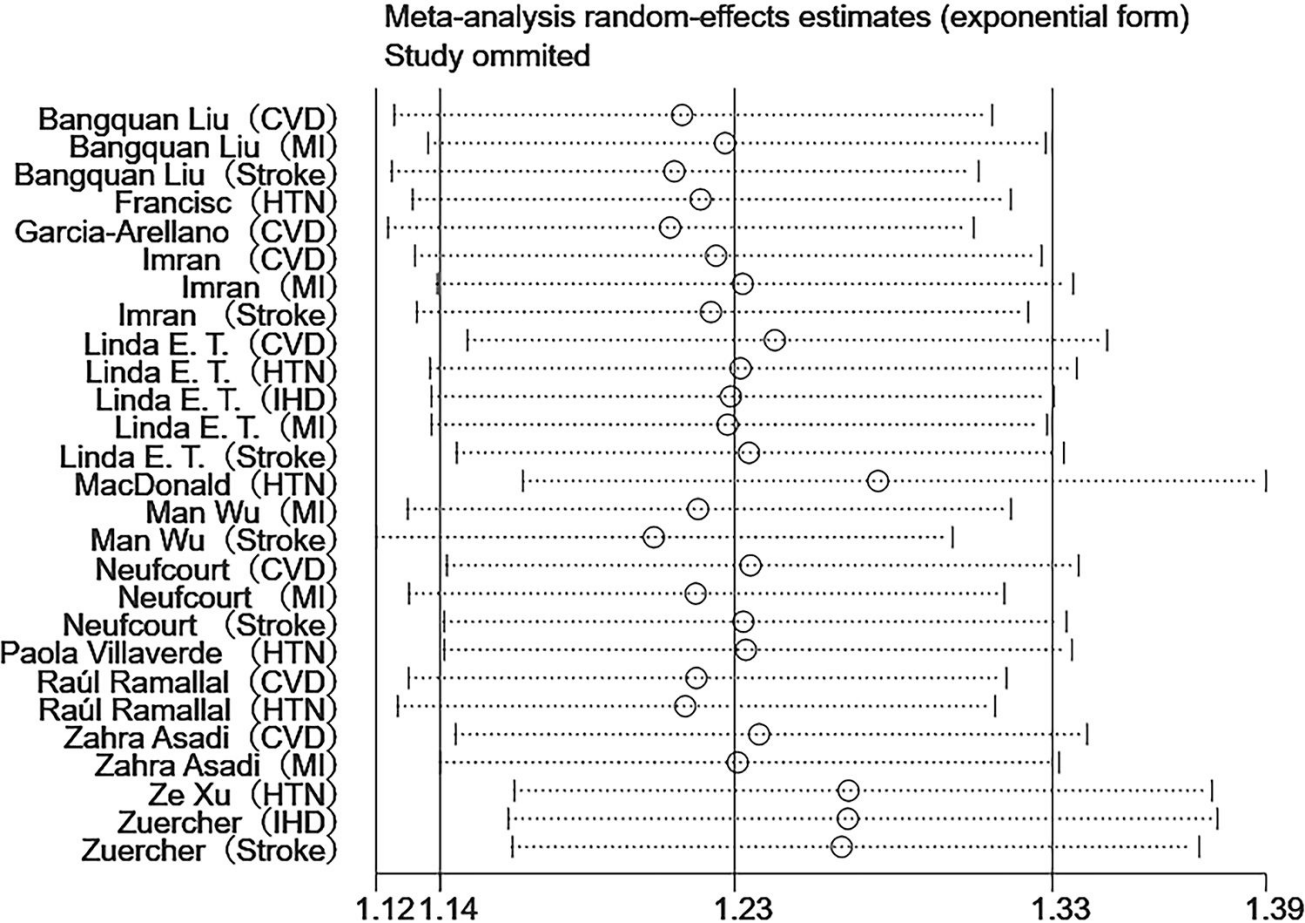
Sensitivity analysis of DII and CVDs risk.

**Figure 5:**
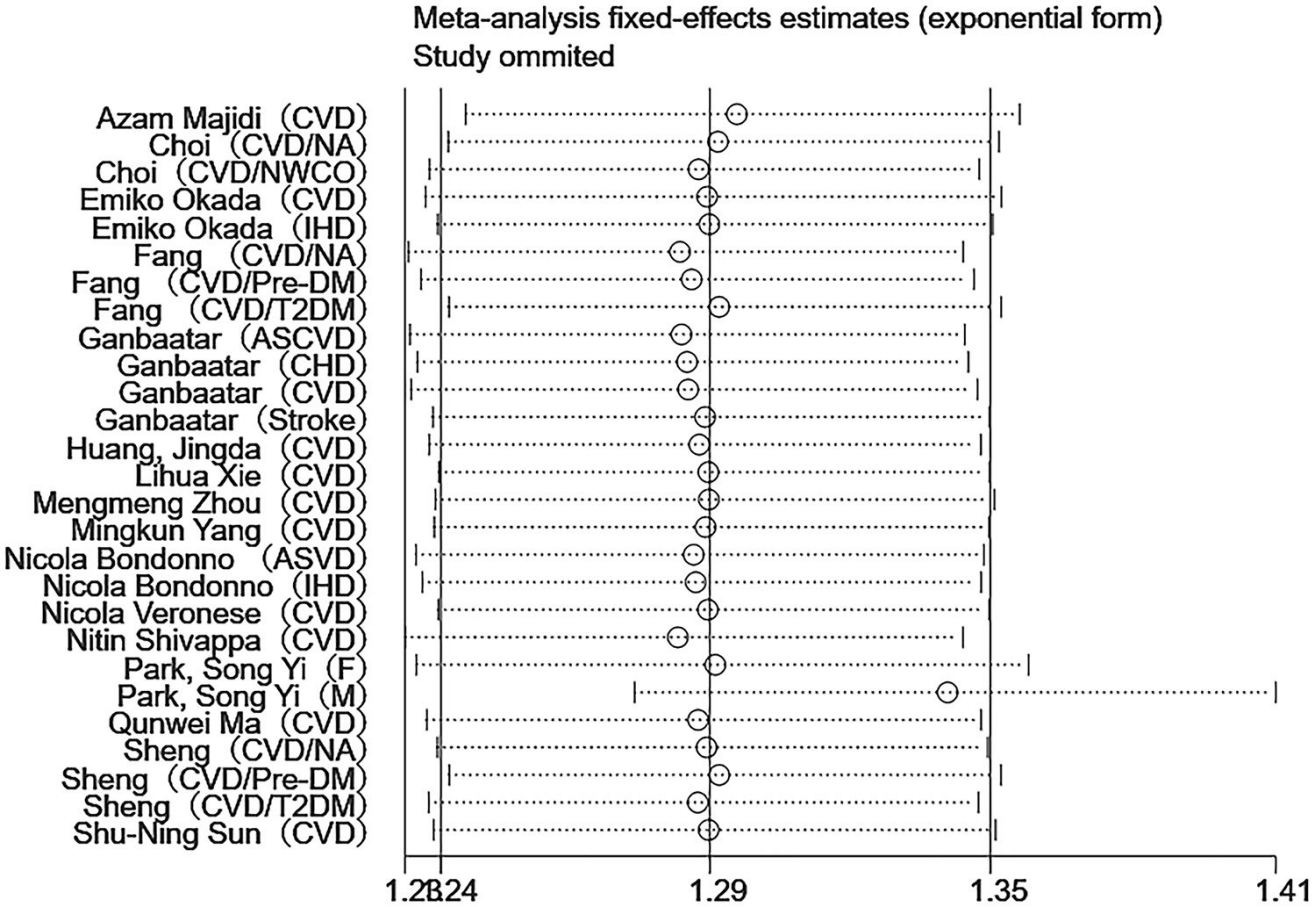
Sensitivity analysis of DII and CVDs mortality.

**Figure 6:**
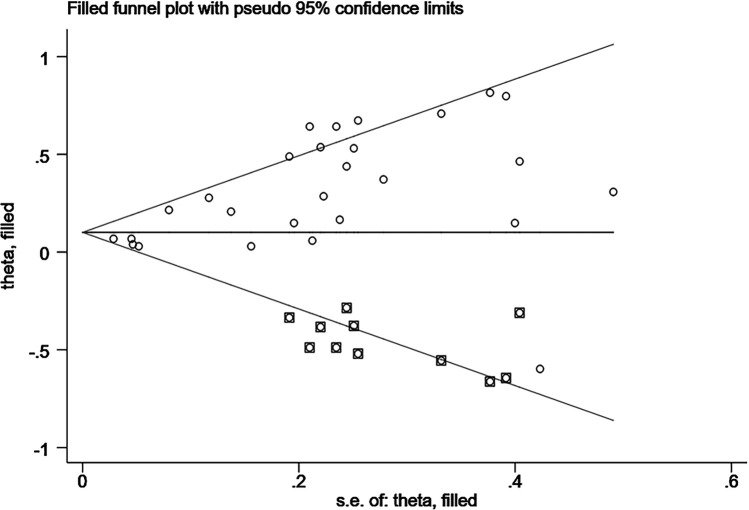
Trim-and-fill funnel plot for the association between DII and CVDs risk.

**Figure 7:**
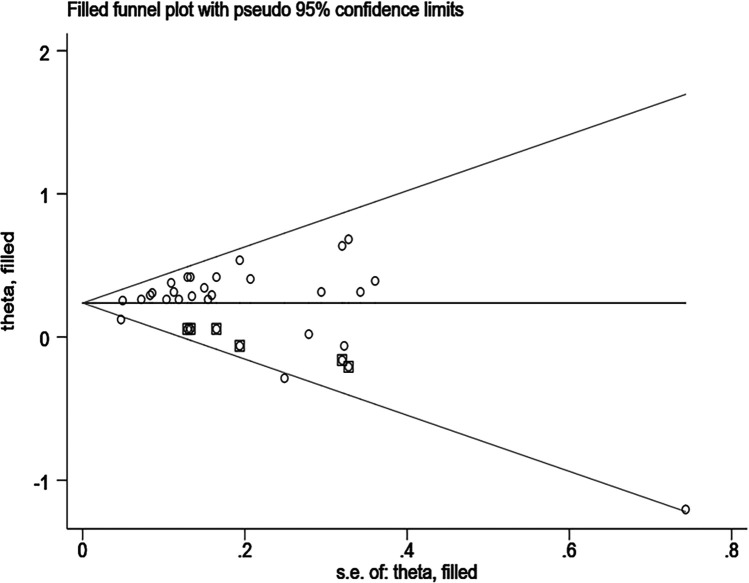
Trim-and-fill funnel plot for the association between DII and CVDs mortality.

Sensitivity analyses were performed using Stata 18.0. The pooled effect estimates for both CVDs incidence and mortality demonstrated minimal changes upon sequential exclusion of individual studies, indicating robust meta-analysis results (Figures 4, 5). Funnel plots revealed asymmetric scatter distributions, suggesting potential publication bias. Trim-and-fill adjustments were subsequently applied. For CVDs incidence analyses, imputation of 11 hypothetical missing studies under a random-effects model yielded a persistent statistically significant association [HR = 1.106, 95% CI (1.018–1.201), *P* < 0.001]. Similarly, imputation of 6 hypothetical missing studies in CVDs mortality analyses under a fixed-effects model maintained significance [HR = 1.268, 95% CI (1.218–1.321), *P* < 0.001], with no reversal in the direction of conclusions. Collectively, these findings confirm the robustness of the meta-analysis results (Figures 6, 7).

## 4 Discussion

This updated systematic review and meta-analysis further substantiates the consistent association between pro-inflammatory dietary patterns, as quantified by the Dietary Inflammatory Index (DII), and elevated risks of cardiovascular disease (CVDs) incidence and mortality. Individuals consuming diets with higher inflammatory potential exhibit significantly greater risks of experiencing CVDs events and related deaths compared to those adhering to diets with lower inflammatory potential. These findings align with a growing body of evidence indicating that unhealthy dietary habits—characterized by excessive intake of processed meats, sugar-sweetened beverages, and refined carbohydrates—adversely affect cardiovascular health (44). Consequently, adopting dietary patterns rich in anti-inflammatory components such as fruits, vegetables, and whole grains may serve as an effective strategy to mitigate the population burden of CVDs (45). In this context, the DII emerges as a practical tool that translates complex nutritional data into actionable indicators of dietary inflammatory potential, enabling clinicians to identify high-risk dietary patterns and provide tailored recommendations to patients.

From a mechanistic perspective, pro-inflammatory diets may promote CVD development through chronic low-grade inflammation and oxidative stress (46). Diets with high DII scores are typically rich in saturated fats, trans fatty acids, and added sugars, which can activate the IKK*β*/NF-*κ*B signaling pathway, stimulating the release of pro-inflammatory cytokines such as interleukin-6 (IL-6) and tumor necrosis factor-alpha (TNF-α) (47). This activation initiates pathogenic cascades leading to atherosclerotic plaque formation, vascular remodeling, and increased arterial stiffness (48). Furthermore, diet-induced inflammatory burden destabilizes the fibrous cap of plaques (49). Evidence suggests that inflammation within the fibrous cap promotes degradation of collagen and extracellular matrix, as well as smooth muscle cell apoptosis, resulting in cap thinning and heightened risk of rupture and thrombosis (50).

In addition, high DII diets are often deficient in antioxidants and phytochemicals, which impairs endogenous defense mechanisms and aggravates oxidative stress–induced vascular injury (51). Insufficient dietary fiber intake also reduces the abundance of butyrate-producing bacteria, thereby lowering short-chain fatty acid (particularly butyrate) production and compromising intestinal barrier integrity. Barrier dysfunction combined with dysbiosis facilitates translocation of gut-derived metabolites such as lipopolysaccharide and trimethylamine (TMA) into the circulation, activating Toll-like receptor 4 (TLR4) and triggering inflammatory cascades that lead to cytokine overproduction and exacerbation of vascular and myocardial injury (52, 53).

Moreover, excessive choline and carnitine in high DII diets are metabolized by the gut microbiota into TMA, which is subsequently oxidized in the liver to trimethylamine-N-oxide (TMAO) (53). This metabolite exerts multiple deleterious effects, including enhancing platelet activation to promote thrombosis, activating the NLRP3 inflammasome to aggravate plaque inflammation, facilitating foam cell formation, and contributing to vulnerable plaque characteristics such as thin fibrous caps and increased microvascularization (54–57). Clinically, TMAO levels independently predict major adverse cardiovascular events (MACE) and adverse prognosis in patients with acute coronary syndrome (58). For example, a meta-analysis by Li et al. demonstrated that each 1 μmol/L increase in TMAO was associated with an ∼11% higher risk of MACE (95% CI: 1.07–1.14; *P* = 0.0000104) (59). Animal studies further revealed that reducing gut-derived metabolites via antibiotic administration significantly attenuated monocyte infiltration and ventricular rupture in myocardial infarction models, supporting a causal role of gut microbiota–mediated inflammation in acute MI (60). Importantly, identical dietary exposures (or equivalent DII scores) do not necessarily result in equal TMAO loads. For instance, microbiota dominated by Firmicutes can substantially enhance TMA-to-TMAO conversion, thereby amplifying pro-inflammatory and pro-thrombotic effects under high DII conditions (61). This provides a biological explanation for interindividual variability in risk responses. Thus, future research on DII–CVD associations should incorporate gut microbiota phenotypes or metabolic capacity into models to better clarify mediating mechanisms and support precision prevention strategies.

Beyond these mechanisms, subgroup analyses in this study indicated potential sex differences in the relationship between dietary inflammation and CVD risk. Elevated DII scores were more strongly associated with CVD incidence among men, whereas the association with CVD mortality was more pronounced among women. This heterogeneity may arise from multiple interacting biological mechanisms. First, estrogen exerts anti-inflammatory and vasoprotective effects. In premenopausal women, estrogen upregulates eNOS expression, enhances nitric oxide production, and suppresses oxidative stress and inflammatory signaling, thereby mitigating vascular injury induced by chronic inflammation (62). However, after menopause, declining estrogen levels weaken this protection, potentially predisposing women to more severe outcomes under high-inflammatory dietary exposure (63). Second, the sex–microbiota–inflammation axis may modulate the strength of DII-related signaling (64). Sex hormones influence microbial composition and metabolic activity, which in turn affect short-chain fatty acid production, intestinal permeability, and endotoxin leakage (65, 66). Under such mechanisms, women may partially buffer pro-inflammatory signaling due to stronger microbial regulatory capacity, whereas men may more readily translate these signals into systemic inflammatory burden. Nonetheless, current evidence is insufficient to conclude that “men are more susceptible to incidence while women are more susceptible to mortality” at equivalent DII levels. Heterogeneity in population composition, follow-up duration, endpoint definitions (incidence vs. mortality), and covariate adjustments across studies complicates interpretation, and most studies lack concurrent assessments of sex hormones, microbiota profiles, inflammatory biomarkers, and sex interactions. Future research should incorporate these mechanistic variables and test sex interactions in larger samples to verify and quantify this heterogeneity.

In addition, methodological subgroup analyses revealed stronger associations in studies using 24HR, studies with energy adjustment, and those without diabetes history adjustment. We interpret these findings as follows: first, compared with FFQ, repeated 24HR captures within-person variation more accurately, thereby reducing non-differential exposure misclassification and biasing effect estimates toward the null (67). Second, energy adjustment “fixes” total energy intake, diminishing confounding from factors such as physical activity and metabolic efficiency, and allowing the analysis to focus on dietary composition–driven inflammatory signals (68). In contrast, adjustment for diabetes history produced weaker associations. This likely reflects the dual role of diabetes history as both a confounder and mediator. Including diabetes as a covariate may block mediating pathways and cause over-adjustment, systematically underestimating the true association (69). However, failure to adjust entirely may leave residual confounding, as diabetes diagnosis and dietary management can alter subsequent DII, while diabetes itself increases CVD risk. Thus, not adjusting could exaggerate or distort the associations. Nevertheless, even in studies adjusting for diabetes, high DII remained positively associated with CVD risk, indicating that DII influences cardiovascular outcomes through mechanisms beyond glycemic pathways, particularly chronic systemic inflammation (70).

Overall, this study demonstrates that pro-inflammatory dietary patterns significantly elevate CVD incidence and mortality, reinforcing the central role of dietary modulation in cardiovascular progression and providing evidence for personalized dietary interventions based on DII. From a practical standpoint, we recommend DII as a supplementary tool for risk stratification and dietary management in addition to traditional CVD risk assessments. Suitable applications include: primary prevention, where baseline nutritional assessments and annual follow-ups are conducted for individuals with multiple cardiometabolic risk factors or family history of early-onset CVD; secondary prevention, where dietary inflammatory load is monitored after hospital discharge and during cardiac rehabilitation follow-up; and clinical decision points such as initiation or intensification of lipid-lowering, glucose-lowering, or weight management interventions, smoking cessation, or exercise prescription. In patients with inflammatory phenotypes or metabolic comorbidities, DII can serve as additional evidence to reinforce lifestyle management. Regarding dietary assessment, repeated 24HR (≥3 non-consecutive recalls, including ≥2 weekdays and ≥1 weekend day) with energy adjustment is recommended for calculating DII (71). In resource-limited settings, simplified FFQs calibrated to local diets may serve as alternatives. For interpretation, DII should be treated as a continuous metric, where negative values indicate relatively anti-inflammatory and positive values relatively pro-inflammatory diets. Given variations in food items, assessment tools, and population characteristics, no universal clinical cutoffs exist. Thus, reporting individuals' percentile rank within a sample, alongside blood pressure, lipids, glucose/HbA1c, anthropometry, and hs-CRP, is a more prudent strategy than using fixed thresholds. Finally, DII interpretation should be linked to heart-healthy dietary patterns and translated into actionable advice. For CVD patients or high-risk individuals, clinicians should encourage increased intake of fruits, dark green leafy vegetables, whole grains, nuts, and *ω*-3–rich fish, preferential use of liquid plant oils, and restriction of red meat, refined sugars, and fried foods (44, 72). Given the positive correlation between DII and CRP, clinicians may consider presenting improvements in DII alongside reductions in inflammatory biomarkers, thereby helping patients recognize the modifiable “diet–inflammation–CVD event” pathway and motivating them to adopt anti-inflammatory dietary habits in daily life.

### 4.1 Strengths and limitations

Compared with previous meta-analyses, this study has several strengths: a larger sample size, broader geographic coverage, and stricter methods for covariate control, publication bias assessment, and robustness testing, all of which enhance the reliability and generalizability of the effect estimates. Nevertheless, some limitations should be acknowledged. First, the lack of age-stratified analyses represents a key limitation. As inflammation and dietary habits vary with age, age may act as an important effect modifier. However, incomplete reporting of mean age and standard deviation in some studies prevented subgroup analyses, potentially underestimating or masking age-related effects. Second, although energy adjustment and multiple covariate controls were applied, residual confounding remains inevitable. Potential unmeasured factors—such as medication use, genetic predisposition, and gut microbiota characteristics—may influence inflammation and cardiovascular outcomes. Third, most included studies were conducted in high-income countries, with limited representation from low- and middle-income countries, restricting the generalizability of findings to diverse socioeconomic and nutritional transition contexts.

Future research should integrate multidimensional information including inflammatory biomarkers, microbiota profiles, and genetic susceptibility to clarify biological mechanisms of dietary inflammatory load and explore differential responses across metabolic states and population subgroups.

## 5 Conclusion

In conclusion, higher Dietary Inflammatory Index (DII) scores are closely associated with increased risks of cardiovascular disease (CVDs) incidence and mortality. Pro-inflammatory diets may accelerate the development of CVDs through systemic inflammation and gut microbiota-mediated pathways. This association may vary by sex and metabolic status, highlighting the importance of nuanced approaches in research and dietary recommendations. Our findings support the promotion of anti-inflammatory dietary patterns as public health measures and clinical interventions to reduce overall cardiovascular risk, advocating for a shift towards more personalized dietary guidance to improve heart health outcomes.

## Data Availability

The original contributions presented in the study are included in the article/Supplementary Material, further inquiries can be directed to the corresponding author.
